# Protein folding prediction in the HP model using ions motion optimization with a greedy algorithm

**DOI:** 10.1186/s13040-018-0176-6

**Published:** 2018-08-08

**Authors:** Cheng-Hong Yang, Kuo-Chuan Wu, Yu-Shiun Lin, Li-Yeh Chuang, Hsueh-Wei Chang

**Affiliations:** 1 Department of Electronic Engineering, National Kaohsiung University of Science and Technology, Kaohsiung, Taiwan; 2 Graduate Institute of Clinical Medicine, Kaohsiung Medical University, Kaohsiung, Taiwan; 3 Department of Computer Science and Information Engineering, National Kaohsiung University of Science and Technology, Kaohsiung, Taiwan; 4 Department of Chemical Engineering & Institute of Biotechnology and Chemical Engineering, I-Shou University, Kaohsiung, Taiwan; 5 Department of Biomedical Science and Environmental Biology, Kaohsiung Medical University, Kaohsiung, Taiwan; 6 Institute of Medical Science and Technology, National Sun Yat-sen University, Kaohsiung, Taiwan; 7 Department of Medical Research, Kaohsiung Medical University Hospital, Kaohsiung, Taiwan

**Keywords:** Protein folding, Ion motion optimization, IMOG, Hydrophobic-polar (HP) model, Global search, Local search

## Abstract

**Background:**

The function of a protein is determined by its native protein structure. Among many protein prediction methods, the Hydrophobic-Polar (HP) model, an ab initio method, simplifies the protein folding prediction process in order to reduce the prediction complexity.

**Results:**

In this study, the ions motion optimization (IMO) algorithm was combined with the greedy algorithm (namely IMOG) and implemented to the HP model for the protein folding prediction based on the 2D-triangular-lattice model. Prediction results showed that the integration method IMOG provided a better prediction efficiency in a HP model. Compared to others, our proposed method turned out as superior in its prediction ability and resilience for most of the test sequences. The efficiency of the proposed method was verified by the prediction results. The global search capability and the ability to escape from the local best solution of IMO combined with a local search (greedy algorithm) to the new algorithm IMOG greatly improve the search for the best solution with reliable protein folding prediction.

**Conclusion:**

Overall, the HP model integrated with IMO and a greedy algorithm as IMOG provides an improved way of protein structure prediction of high stability, high efficiency, and outstanding performance.

## Background

Polypeptides consist of a maximum of 20 amino acids. The function of a given protein is determined by the native structure or its polymer structure, which correlates with particular protein functions [1]. The native three-dimensional structure of a protein primarily depends on its amino acid sequence [2]. The development of a highly efficient method for protein folding prediction is in high demand, particularly for protein studies in biotechnology. Currently, several methods have been proposed for protein structure prediction. Comparative modeling and fold recognition approaches commonly use a known protein structure database to train a model in order to classify an unknown protein structure [2]. In contrast, the ab initio method provides a direct prediction using the primary structure or amino acid sequence of a given protein.

Based on the ab initio method, Dill has proposed the hydrophobic-polar protein folding model (HP model) in 1985 which simulates protein folding based on amino acid sequences under the lattice model [3]. In 2013, Bechini used a triangular lattice model for protein folding prediction by simplifying amino acids into hydrophobic (H) and polar (P) types [4]. The predicted space in between the simulated folds is limited to the lattice model; the actual fold space is discrete and the folding of the amino acid sequence follows a self-avoiding walk along the lattice. However, for the protein structure prediction, it remains a challenge to explore the possibility of an extremely large folding in order to obtain an optimal solution for the nondeterministic polynomial-time-hard (NP-hard) problem [5].

Anfinsen’s dogma, following a thermodynamic hypothesis, assumes that the native structure of globular proteins usually folds according to a unique, stable, and kinetically accessible minimum of free energy [6]. The central structure of a globular protein usually contains hydrophobic (non-polar) amino acid compositions that produce hydrophobic attraction to avoid water molecules at the outside. This postulate was also applied to the HP model-based protein structure prediction. To provide an example, if two hydrophobic amino acids are closed together, a hydrophobic-hydrophobic (H-H) interaction is generated. Once the strength of H-H interactions is increased, a more stable structure is predicted. The HP model uses this property giving a negative value for the adjacent hydrophobic amino acids interaction and calculating the number of adjacent hydrophobic interactions [7]. When more adjacent hydrophobic amino acids are present, the predicted structure is closer to the real structure representing optimal protein folding. However, the development of an optimal algorithm for protein folding prediction remains a challenge.

A 2D-triangle-lattice-model [8] is commonly used for 2D HP model of protein folding problem. It has six neighbors in the two dimension triangular lattice on each lattice. When a triangle-lattice-model is embedded in the protein, it can be in topological contact with each other. The vector obtained from the triangle lattice that is easier to model a protein structure on the 2D-triangle-lattice-model [9]. The self-avoiding walk for protein folding is the NP-hard problem and led to several heuristic and meta-heuristic algorithms that were proposed to find best protein structure predictions. These include among others: genetic algorithm [10], branch and bound [11], replica exchange Monte Carlo [12], Evolutionary Monte Carlo [13], greedy-like-algorithm [14].

We use here an ions motion optimization (IMO) algorithm [15] as a heuristic algorithm that is combined – as a novum – with a greedy algorithm for local search within the 2D-triangle-lattice-model to optimize protein folding predictions of high stability, high efficiency, and outstanding performance.

## Methods

We develop here a novel algorithm (IMOG) which combines ions motion optimization (IMO) algorithm [15] with a greedy algorithm as a local search strategy for predicting protein folding reliably at high resolution. Details of our approach are described below.

### Protein folding problem of a 2D-HP-model

The HP-model is a well-known process for protein folding simulation that simulates the hydrophobic interaction between amino acid residues. All 20 amino acids are classified into two groups based on their hydrophobicity/hydrophilicity: H represents hydrophobic/nonpolar residues and P represents hydrophilic/polar residues. Given *N* amino acids residue sequences of a protein chain, S = {*a*_1_, *a*_2_, …, *a*_*N*_} ⊂ {H, P}. The energy *E* of a chain conformation was proposed for comparing the stability of several predicted protein structures [7]. The energy *E* of a chain conformation was defined to the number of H-H contacts *h*, i.e., *E* = − |ε| *h*, where |ε| is a positive constant. The units of energy *E* is |ε|. For simplification, the energy *E* of protein conformation is calculated by the following formula as described previously [9, 10]:

1 $$ E={\Sigma}_{i,j}\varDelta {r}_{ij}{\in}_{ij} $$

where

2 $$ {\in}_{ij}=\left\{\begin{array}{l}-1.0, the\ pair\ of\ H\  and\ H\  residues\\ {}0.0, others\end{array}\right. $$

and

3 $$ \varDelta {r}_{ij}=\left\{\begin{array}{l}1, if\;{a}_i\;\mathrm{and}\;{a}_j\;\mathrm{are}\;\mathrm{adjacent}\;\mathrm{but}\;\mathrm{do}\;\mathrm{not}\kern0.17em \mathrm{connect}\kern0.17em \mathrm{amino}\kern0.17em \mathrm{acids}\\ {}0, others\end{array}\right. $$

The 2D-HP protein folding problem can be formally defined as finding a conformation of S with minimum energy. This has been proven to be a NP-hard problem.

### Imo

The IMO algorithm has been recently introduced as a metaheuristic optimization technique and it is inspired by the motion of ions [15]. Each single ion in its particular position provides a candidate for solving a particular optimization problem. The movements of ions depend on the attraction or repulsion of ions, i.e., anion (negative charged ion) and cation (positive charged ion). The attraction and repulsion forces between anions and cations are utilized to move the position of ions around a feasible search region. The forces are calculated as acceleration of ion motions. Anions move towards a best fitness of cations and cations move towards a best fitness of anions. Two strategies of ion motion for providing diversification and intensification, these are movements in a liquid phase and a solid phase scenario, respectively. In this study, we implemented an IMO as a heuristic algorithm to find the best HP model for protein folding simulation.

In the 2D-dimensional search space, assuming a population consists of *N* anions/cations moving around. The *i*th anion and *i*th cation are represented by *A*_*i*_ = (*a*_*i*1_, *a*_*i*2_, …, *a*_*iD*_) and *C*_*i*_ = (*c*_*i*1_, *c*_*i*2_, …, *c*_*iD*_) in their respective position. The populations of anions and cations are initialized by a uniform random position ***A*** ∈ {*A*_1_, *A*_2_, ..., A_*N*_} and position ***C*** ∈ {*C*_1_, *C*_2_, ..., *C*_*N*_}. Each position of an ion provides a candidate solution for a particular problem. When the fitness of evaluation results is calculated, the global best solutions (*Abest* and *Cbest*) and current individual worst solutions (*A*_*worst*_ and *C*_*worst*_) are determined.

In the liquid phase strategy, the attraction forces are used for a search space [15], and its computation is calculated from the distance between two ions (e.g., anion and cation), the measurement is defined as follows:

4 $$ {AF}_{i,j}=\frac{1}{1+{e}^{-0.1/{AD}_{i,j}}} $$

5 $$ {CF}_{i,j}=\frac{1}{1+{e}^{-0.1/{CD}_{i,j}}} $$

where *AD*_*i*, *j*_ = |*A*_*i*, *j*_ − *Cbest*_*j*_| is the distance between the current anion position and the globally best cation position; *CD*_*i*, *j*_ = |*C*_*i*, *j*_ − *Abest*_*j*_| is the distance between the current cation position and the globally best anion position. *AF*_*i*,*j*_ and *CF*_*i*,*j*_ represent resultant attraction forces of anions and cations, respectively.

According to formula (4) and (5), the position of anions and cations based on the attraction force are updated as the following equations:

6 $$ {A}_{i,j}={A}_{i,j}+{AF}_{i,j}\times \left( Cbes{\mathrm{t}}_j-{A}_{i,j}\right) $$

7 $$ {C}_{i,j}={C}_{i,j}+{CF}_{i,j}\times \left({Abest}_j-{C}_{i,j}\right) $$

In the solid phase strategy, when convergence has occurred or solid phase conditions are satisfied, external forces are evaluated in order to escape entrapment in the local optima, and the formula is described as follows:

8 $$ {\displaystyle \begin{array}{l}\boldsymbol{if}\; CbestFit\ge CworstFit/2\ \mathrm{and}\  AbestFit\ge AworstFit/2\\ {}\kern1.08em \boldsymbol{if}\;{\operatorname{rand}}_1\left(\;\right)>0.5\\ {}\kern2.04em {A}_i={A}_i+{\Phi}_1\times \left( Cbest-1\right)\\ {}\begin{array}{l}\kern1.08em \boldsymbol{else}\\ {}\kern2.04em {A}_i={A}_i+{\Phi}_1\times Cbest\\ {}\kern1.08em \boldsymbol{end}\;\boldsymbol{if}\\ {}\begin{array}{l}\kern1.08em \boldsymbol{if}\;{\operatorname{rand}}_2\left(\;\right)>0.5\\ {}\kern2.04em {C}_i={C}_i+{\Phi}_2\times \left( Abest-1\right)\\ {}\kern1.08em \boldsymbol{else}\\ {}\begin{array}{l}\kern2.04em {C}_i={C}_i+{\Phi}_2\times Abest\\ {}\kern1.08em \boldsymbol{end}\;\boldsymbol{if}\\ {}\kern1.08em \boldsymbol{if}\;{\operatorname{rand}}_3\left(\;\right)<0.05\\ {}\begin{array}{l}\kern1.92em \operatorname{Re}\hbox{-} \mathrm{initialized}\;{A}_i\ \mathrm{and}\ {C}_i\;\mathrm{with}\kern0.17em \mathrm{random}\kern0.17em \mathrm{position}\\ {}\kern1.08em \boldsymbol{end}\;\boldsymbol{if}\\ {}\boldsymbol{end}\;\boldsymbol{if}\end{array}\end{array}\end{array}\end{array}\end{array}} $$

where Φ_1_ and Φ_2_ are random numbers in the range of −1 to 1. *rand*_1_(), *rand*_2_() and *rand*_3_() are random numbers in the range of 0 to 1. *AworstFit* and *CworstFit* are the worst fitness solutions of anion and cation fits, respectively.

### Greedy algorithm

The greedy algorithm is a simple and straightforward heuristic algorithm that makes a current local optimal decision at each stage for global optimization [16]. It is easy to implement and works efficiently depending on the problems although it may or may not be the best approach for solving this task. In any case, it plays a useful role as a optimization method according to its characteristics. Greedy algorithms are widely applied in bioinformatics tools such as among others DNA sequence alignment [16], co-phylogeny reconstruction problem [17], detection of transient calcium signaling [18], resolving the structure and dynamics of biological networks [19].

### IMOG for 2D-HP-model

We implemented the IMO algorithm with a greedy algorithm as a local search strategy for the 2D-HP-model protein folding problem as follows (including IMOG procedure, encoding scheme, fitness function, and improved solid phase strategy):

#### IMOG procedure

**Fig. 1:**
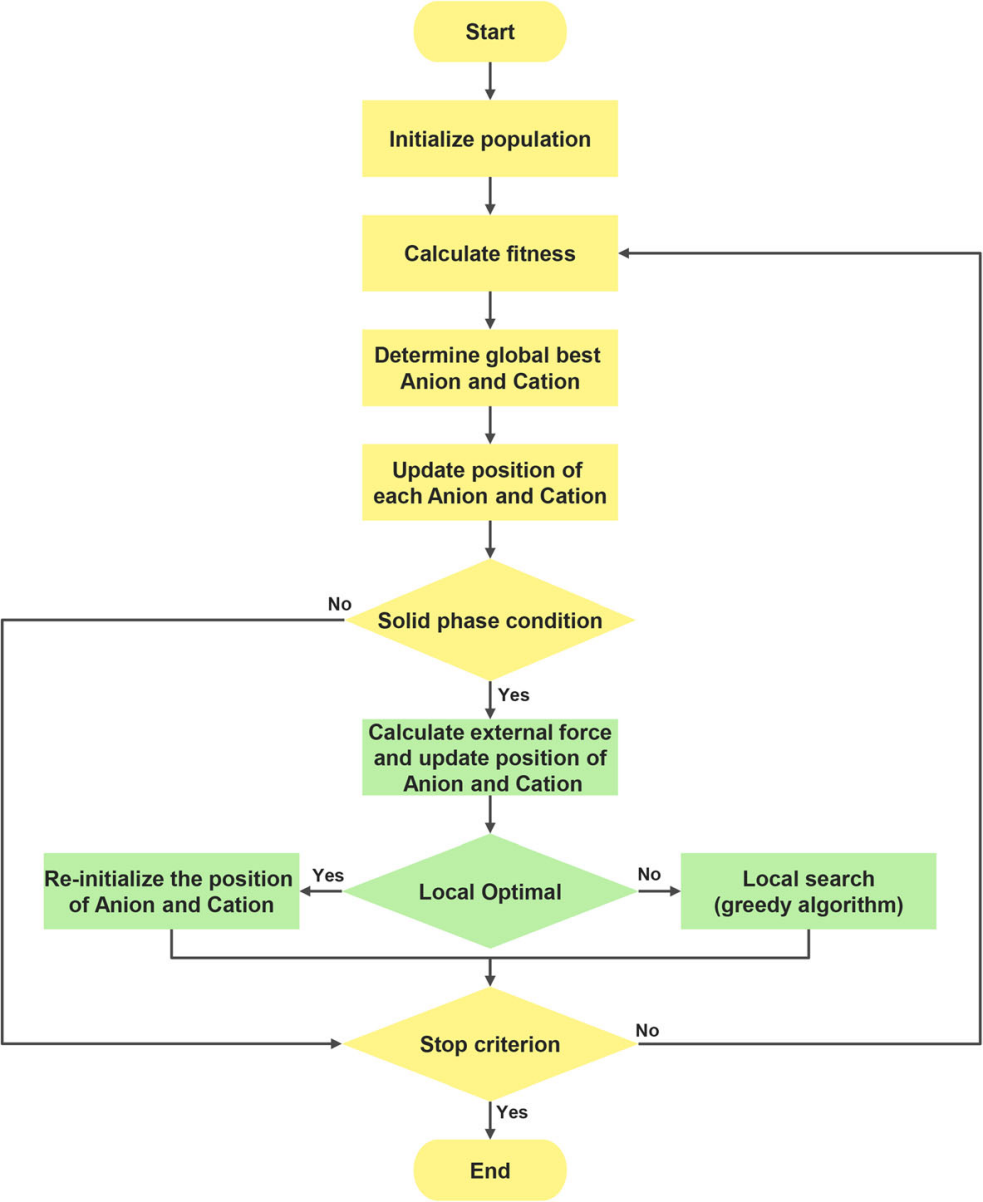
Flowchart for developing an IMO algorithm

This study presents an improved IMO with a greedy algorithm to be implemented in a 2D-HP-model process. The flowchart of our proposed method is shown in Fig. 1, and the detailed procedure of IMOG is described as follows:

Step 1) Initialize populations of ions (anions and cations) with random position, each position of an ion is a candidate for the protein folding.
Step 2) Estimate the fitness of each ion using energy of the 2D-HP-model according to the Eq. (9).
Step 3) Update the global best solution *Abest* and *Cbest* according to the fitness calculation results.
Step 4) Calculate the force and update position of each ion according to the Eqs. (4)–(7).
Step 5) If the solid phase condition was satisfied, the solid phase strategies were executed.
Step 6) Repeat steps 2–5 until the stop criterion has been met. Consequently, the best protein folding was obtained.

#### Encoding scheme

**Fig. 2:**
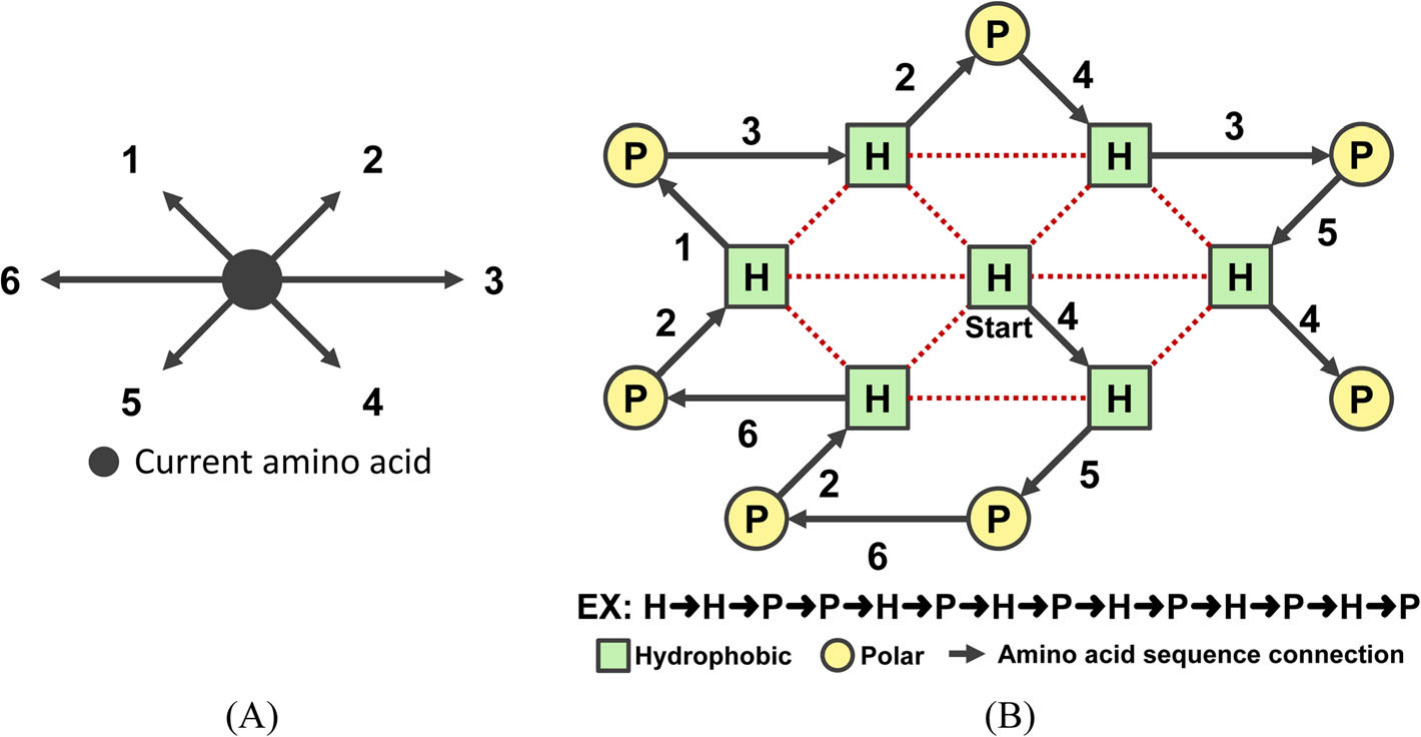
Example of a 2D-HP-model. **a** 2D triangular lattice model with six neighbors. **b** Illustration of best solution in 2D-HP-model. The arrow with number indicates next direction of current amino acid. The red dotted line is H-H contact

In accordance with the previous description of problems, we utilized the integer IMO to proceed with the analysis. The neighbors of vertex in the 2D-triangular-lattice model in IMO are under anion/cation coding. Each position of anion/cation was designed in a format as the amino acid directions of movement in protein folding, and this was described as *A*_*i*_ = (*d*_*i*1_, *d*_*i*2_, …, *d*_*iD*_) and *C*_*i*_ = (*d*_*i*1_, *d*_*i*2_, …, *d*_*iD*_), respectively, where *d* ∈ {1, 2, …, 6} represents six neighbors in the 2D-triangular-lattice-model (Fig. 2a). For example, the IMO encoding the best solution for the sequence HHPPHPHPHPHPHP presents as 4, 5, 6, 2, 6, 2, 1, 3, 2, 4, 3, 5, and 4 (Fig. 2b).

#### Fitness function

Two hydrophobic amino acids *x*_*i*_ and *x*_*j*_ of the lattice positions *p*_*i*_ and *p*_*j*_ are respectively indicated to have a H-H contact that is represented as *contact*(*p*_*i*_, *p*_*j*_) = − 1, otherwise *contact*(*p*_*i*_, *p*_j_) = 0. The energy of the protein conformation is defined as the sum of its H-H contacts. If *S* has an HP sequence, and *P* = *p*_1_, *p*_2_, ..., *p*_*n*_ represents a valid conformation for *S*, the energy *E*(*P*) of *P* is defined as follows:

9 $$ fitness={\Sigma}_{i=1}^{n-2}{\Sigma}_{j=i+2}^n contact\left({p}_i,{p}_j\right) $$

**Fig. 3:**
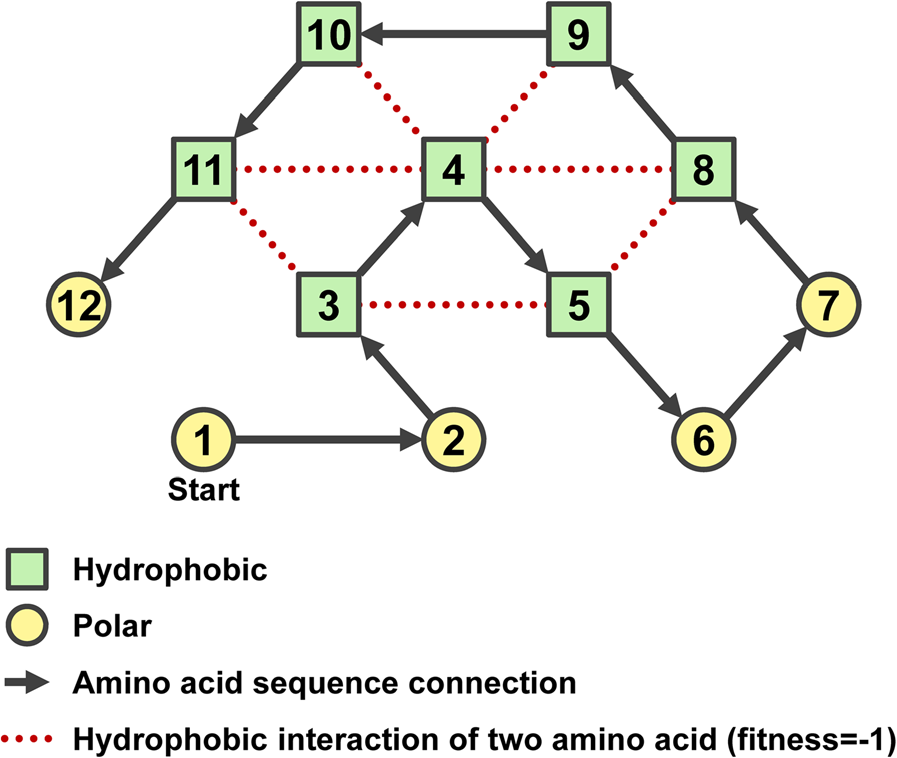
Illustration of the best fit obtained from the 2D-HP-model

In the case of Fig. 3, the point *p*_4_ is not adjacent to *p*_8_, *p*_9_, *p*_10_ and *p*_11_ in the amino acid chain but is adjacent in the 2D-model space, thus the fitness is calculated as − 4. Consequently, (*p*_3_, *p*_5_), (*p*_3_, *p*_11_) and (*p*_5_, *p*_8_) are estimated as − 3, non-repetitively. Taken together, the fitness as the energy is − 7.

#### Improved solid phase strategy

The original condition of solid phase (e.g., if *CbestFit* ≥ *CworstFit* / 2 AND *AbestFit* ≥ *AworstFit* / 2) is unsuitable for our problem. Therefore, we improve the solid phase statement for the HP-model protein folding prediction. In order to enhance the optimal solution, we add the local search method to this phase and pseudo-codes are provided in the formula (10).

10 $$ {\displaystyle \begin{array}{l}\boldsymbol{if}\; CbestFitNum\ \mathrm{and}\  AbestFitNum\;\mathrm{do}\ \mathrm{not}\kern0.17em \mathrm{change}> SolidNum\\ {}\kern0.96em \boldsymbol{if}\;{\operatorname{rand}}_1\;\left(\;\right)>0.5\\ {}\kern2.04em {A}_i={A}_i+{\Phi}_1\times \left( Cbest-1\right)\\ {}\kern0.96em \boldsymbol{else}\\ {}\kern2.04em {A}_i={A}_i+{\Phi}_1\times Cbest\\ {}\kern0.96em \boldsymbol{end}\;\boldsymbol{if}\\ {}\kern0.96em \boldsymbol{if}\;{\operatorname{rand}}_2\left(\;\right)>0.5\\ {}\kern1.92em {C}_i={C}_i+{\Phi}_2\times \left( Abest-1\right)\\ {}\kern0.96em \boldsymbol{else}\\ {}\kern1.92em {C}_i={C}_i+{\Phi}_2\times Abest\\ {}\kern0.96em \boldsymbol{end}\;\boldsymbol{if}\\ {}\kern0.96em \boldsymbol{if}\;{\operatorname{rand}}_3\left(\;\right)<0.05\\ {}\kern1.8em \mathrm{Local}\kern0.17em \mathrm{search}\hbox{-} \mathrm{greedy}\kern0.17em \mathrm{algorithm}\\ {}\kern0.96em \boldsymbol{else}\\ {}\kern1.8em \operatorname{Re}-\mathrm{initialized}\;{A}_i\ \mathrm{and}\ {C}_i\;\mathrm{with}\kern0.17em \mathrm{random}\kern0.17em \mathrm{position}\;\\ {}\kern0.96em \boldsymbol{end}\;\boldsymbol{if}\\ {}\boldsymbol{end}\;\boldsymbol{if}\end{array}} $$

where *CbestFitNum* and *AbestFitNum* are the numbers for the global best solution of cation/anion not yet changed. *SolidNum* is a parameter setting for how many times the *CbestFitNum* and *AbestFitNum* were not yet changed.

**Fig. 4:**
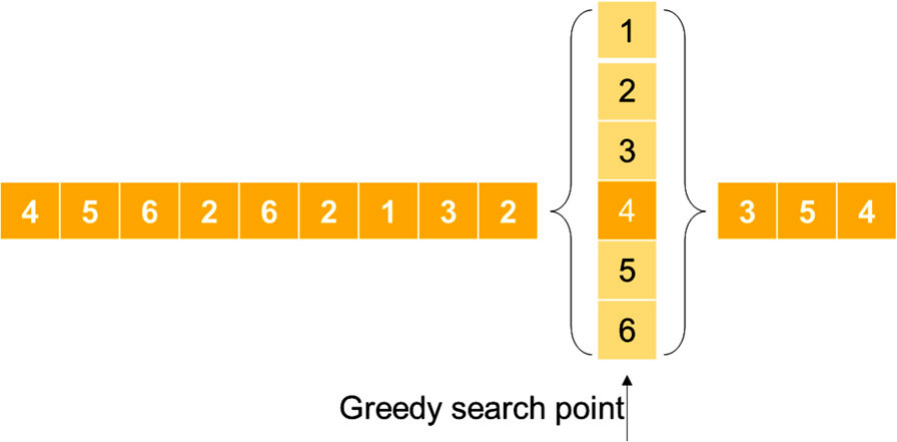
Illustration of IMO with a local search by a greedy algorithm. The greedy algorithm is utilized for local search that randomly selects the position point and searches the best fitness of six neighbors

In this study, the greedy algorithm is utilized for a local search that randomly selects the point position of an anion/cation and to search six neighbors for their best solution. In the sequence HHPPHPHPHPHPHP example, through the greedy algorithm the best solution for the tenth point is 4. This is illustrated in Fig. 4.

## Results

### Data sets

In the current study, two benchmark data sets for the 2D HP model protein prediction are used and the details are shown in Tables 1 and 2. These tables show the information of amino acid sequences including sequence number, length, sequence, and optimal known energy values. The sequences are described as binary symbols where 0 indicates the polar residues and 1 indicates the hydrophobic residues. The data within parenthesis (…)_*i*_ represent the *i*-fold duplicate of a subsequence. For example, (010)_2_ represents the original information of data being 010010 (e.g., HPHHPH). Recently, several approaches such as hybrid of hill climbing and genetic algorithm [9], elite-based reproduction strategy-genetic algorithm (ERS-GA) [9], cached divide and conquer evolutionary algorithm [20], co-evolution of memetic algorithms [21], hybrid genetic algorithm (HGA) [22], and tabu search (TS) [23] have been proposed to predict protein folding using benchmark data sets, through HP-model-based computation estimates for protein structure prediction.

**Table 1:** First benchmark of amino acids sequences in HP model [9]

Sequence	length	***E*** ^*1^	Amino acids sequence^*2^
1	20	-15	(101001)_2_0110(01)_2_
2	24	−17	1(100)_2_1(001)_5_1
3	25	−12	(001)_2_(100001)_3_1
4	36	−24	0(0011)_2_(0)_5_(1)_7_(001100)_2_100
5	48	−43	001(0011)_2_(0)_5_(1)_10_(0)_6_(1100)_2_100(1)_5_
6	50	−41	1(10)_4_(1)_4_(0100)_3_00(1000)_2_10111(10)_4_11
7	60	–	001110(1)_8_000(1)_10_01000(1)_12_(0)_4_(1)_4_011010
8	64	–	(1)_12_(01)_2_0(1100)_2_(1001)_2_(100)_2_(1100)_2_(10)_2_(1)_12_

^*1^***E*** is the best energy [7] value in 2D HP model. *E* was defined to the number of H-H contacts *h*, i.e., *E* = − |ε| *h*, where |ε| is a positive constant. The units of energy *E* is |ε|. For simplification, *E* is calculated by the following formula as described previously [9, 10] as mentioned in formula (1) to (3)

^*2^ 0 represents hydrophobic (H); 1 represents polarity (P) in amino acids sequence; (…)_*i*_ represents *i*-fold repetitions of the respective subsequence in data

**Table 2:** Second benchmark of amino acids sequences in HP model [21]

Sequence	length	***E*** ^*1^	Amino acids sequence^*2^
1	12	−11	1(10)_5_1
2	14	−11	1100(10)_5_
3	14	−11	1(100)_2_(10)_3_1
4	16	−11	110(100)_4_1
5	16	−11	1(100)_2_(10)_3_010
6	17	−11	1(100)_5_1
7	17	−17	1(11)_7_11
8	20	− 17	1(100)_2_(10)_3_(01)_3_1
9	20	−17	1(10)_4_1(001)_3_1
10	21	−17	1(100)_2_(10100)_2_1011
11	21	−17	110(100)_2_(10)_2_(100)_2_11
12	21	−17	1100(10)_3_(01)_2_(001)_2_1
13	22	−17	1(100)_2_(10)_3_(010)_2_011
14	23	−25	11(10)_9_111
15	24	−17	1(100)_7_11
16	24	−25	11(10)_3_(01)_7_11
17	24	−25	11(10)_4_(01)_6_11
18	30	−25	11(100)_4_1(01001)_2_00111
19	30	−25	11(100)_3_(10)_2_(01)_2_(001)_3_11
20	37	−29	11(100)_3_(10)_2_1(001)_3_(0)_5_(10)_2_111

^*1^***E*** is the best energy value in 2D HP model. *E* is calculated by the following formula as described previously [9, 10] as mentioned in formula (1) to (3)

^*2^0 represents hydrophobic (H); 1 represents polarity (P) in amino acids sequence; (…)_*i*_ represents *i*-fold repetitions of the respective subsequence in data

### Parameter settings

The advantages of the IMOG algorithm is a fewer number of tuning parameters, only population size (e.g., number of anion and cation) and iteration size. Here, we set 100 for the numbers of anions and cations and 2000 for the iteration size in this study. Additionally, the mutation probability of a greedy algorithm for each point in the local search is 0.25.

### Comparison of the best prediction

Table 3 shows a comparison of the best optimal solution obtained from approaches taken from the HGA [22], TS [23], ERS-GA [9], HHGA [9], and our proposed IMOG algorithms. Table 4 compares MMA [24] (Multimeme algorithms for protein structure prediction) with our proposed IMOG approach for predicting results. These methods were applied to eight and twenty amino acids sequence data sets, respectively, that were independently run 30 times for each experiment. All methods can easily obtain the best solution when the length is smaller than 20. However, our proposed method can even obtain best solution when the length is smaller than 40. Table 3 shows the TS is better than our proposed IMOG method for the sequence 7 and HHGA is better than ours for the sequence 5. Nevertheless, IMOG obtains outstanding results for all eight sequences, especially the longest one. Table 4 shows that IMOG can obtain the best outcomes for all 20 sequences. In contrast, IMOG cannot obtain an optimal solution when the sequence length is longer than 30. The results show that the IMOG method proposed here predicts protein folding structure better than other available methods.

**Table 3:** Comparison of algorithms studied here for optimal solutions

Sequence^*1^	SGA	HGA	TS	ERS-GA	HHGA	IMOG
1	−11	**−15**	**−15**	**−15**	**− 15**	**−15**
2	−10	−13	**−17**	− 13	**− 17**	**−17**
3	− 10	− 10	**− 12**	−12	**− 12**	**−12**
4	− 16	−19	**− 24**	− 20	− 23	**− 24**
5	−26	−32	− 40	− 32	**− 41**	−40
6	−21	−23	NA	−30	−38	**−40**
7	−40	− 46	**− 70**	− 55	− 66	− 67
8	− 33	−46	−50	− 47	− 63	**− 69**

^*1^Sequences from Table 1. Bold numbers indicate the best solution for the same test sequence

NA: not available; SGA: simple genetic algorithm; HGA: Hybrid Genetic Algorithm [22]; TS: tabu search [23]; ERS-GA: elite-based reproduction strategy-genetic algorithm [9]; HHGA: hybrid of hill climbing and genetic algorithm [9]; IMOG: Ions motion optimization with a greedy algorithm

**Table 4:** Comparison of the best prediction results of IMO with MMA algorithm

Sequence*^1^	MMA	IMOG	Sequence*^1^	MMA	IMOG
1	NA	**−11**	11	**−17**	**−17**
2	**−11**	**−11**	12	**−17**	**−17**
3	**−11**	**−11**	13	**−17**	**−17**
4	**−11**	**−11**	14	**−25**	**−25**
5	**−11**	**−11**	15	−16	**−17**
6	**−11**	**−11**	16	**−25**	**− 25**
7	**−17**	**−17**	17	**−25**	**−25**
8	**−17**	**−17**	18	−24	**−25**
9	**−17**	**−17**	19	−24	**−25**
10	**−17**	**−17**	20	−26	**−29**

^*1^. Sequences from Table 2. Bold number indicates the best solution for the same test sequence

*NA* not available, *MMA* Multimeme Algorithm using the new mating strategy based on the contact map memory [24], *IMOG* Ions motion optimization with greedy algorithm

### Comparsion of stability

Table 5 shows the comparison of the stability of algorithm outcome utilizing the optimal results and means in 30 independent runs. The literature of MMA did not provide the results for stability analysis, hence the outcome of IMOG was unable to compare with MMA. The optimal results and means can be observed the ability of algorithm finding best structure and the stability of algorithm. Table 5 shows that the mean of fitness of IMOG is better than that of ERS-GA and HHGA in each sequence significantly although the best fitness of HHGA is slightly better than IMOG. Consequently, the result indicates that the IMOG had a high quality in searching stability.

**Table 5:** Comparison of the best solutions and stabilities with other algorithms

Sequence*^1^	***E*** ^*2^	ERS-GA		HHGA		IMOG	
		Best	Mean	Best	Mean	Best	Mean
1	−15	**−15**	−12.50	**−15**	−14.73	**− 15**	− 14.73
2	− 17	− 13	−10.20	**−17**	−14.93	**− 17**	−14.93
3	− 12	**− 12**	− 8.47	**− 12**	− 11.57	**−12**	− 11.57
4	− 24	−20	− 16.17	−23	−21.27	− 23	−21.27
5	−43	−32	−28.13	**−41**	− 37.30	**− 41**	− 37.30
6	−41	− 30	− 25.30	−38	−34.10	− 38	−34.10
7	–	−55	−49.43	− 66	− 61.83	− 66	−61.83
8	–	−47	−42.37	−63	− 56.53	−63	−56.53

^*1^Sequences from Table 1. Bold number indicates the best solution for the same test sequence

^*2^***E*** is the best energy value in 2D HP model. *E* is calculated by the following formula as described previously [9, 10] as mentioned in formula (1) to (3)

*ERS-GA* elite-based reproduction strategy-genetic algorithm [9], *HHGA* hybrid of hill climbing and genetic algorithm [9]; *IMOG* Ions motion optimization with greedy algorithm

## Discussion

The IMO algorithm [15] is a population-based algorithm designed according to the natural properties of ions. Its idea is to divide the ion population into negative and positive charged ions (i.e., anions and cations). It is based on the fact that anions repel anions but attract cations and cations repel cations but attracts anions. It is reported that IMO is very competitive in solving challenging optimization problems [15]. Moreover, the greedy algorithm is also reported to improve local searches [25]. In computer science, hybrid algorithms are commonly applied in solving optimization problems [26–32]. Accordingly, we developed a novel algorithm that combines the IMO algorithm [15] with a greedy algorithm we here name IMOG for protein folding prediction. The key concept of our proposed IMOG algorithm is based on the characteristics of IMO having global search capabilities while escaping from the local best solution. In addition, the greedy algorithm is used in each update to strengthen its local search ability.

In this paper, two phases (liquid and solid) were designed for diverse and intense search that can make sure convergence of the ions toward an optimum in the feasible space and resolve local optima trap. Our proposed method has redundant extra parameters and it adapts itself automatically to search spaces. The obtained results indicate that the integrated algorithm has a good search ability and stability. Compared with other methods, the stability and search ability of our proposed method is better than other methods for protein structure prediction for most of the test sequences.

The HP-model of protein structure prediction problem was developed as discrete problem in folding space. In HP model, the amino acids were classified into hydrophobic and polar that keeps the prediction complexity down. Nevertheless, the whole possible combinations of protein folding prediction problem is still complex. Recently, researchers assume that the simple optimization algorithms were hard to solve protein folding structure prediction effectively [21]. Accordingly, many improved algorithms were proposed to enhance ability of prediction in HP model problem, such as HHGA [9]. The HHGA is an effective algorithm which combines genetic algorithm with a hill climbing algorithm, it can solve longer amino acid sequence well performance.

In this study, we implement an IMO with a greedy algorithm as local search for a 2D-HP model protein folding problem. The technical behavior (liquid phase strategy) of IMO is similar to the particle swarm optimization (PSO) [33] algorithm but the IMO had improved the “particle” to divide into two parts as anion and cation. The two global superior solutions were utilized to search global optimal solutions. It also had a mechanism to escape local optima through the solid phase strategy. We improved the IMO in order to enhance seeking local optima by adding a greedy algorithm to the solid phase strategy. Consequently, our proposed IMOG algorithm has several advantages including low computational complexity, rapid convergence, a smaller number of tuning parameters, avoidance of local optima and superior performance in searching for global optima [15].

Recently, several protein structure prediction systems were developed. For example, Rosetta [34–36] and i-TASSER [37, 38] are sophisticated comprehensive software suites for protein structure and function prediction. Structure prediction with Rosetta was reported to be enhanced performance with an additional modeling, such as the combined covalent-electrostatic model of hydrogen bonding [34]. The processing that generates protein structure and function predictions by i-TASSER is firstly retrieved from protein data bank (PDB) library by Local Meta-Threading-Server (LOMETS) [39]. When LOMETS is unable to identify suitable template, i-TASSER will process the ab initio modeling for protein structure and identify the low free energy states by SPICKER [40]. It is possible that our proposed IMO may support the function of SPICKER and i-TASSER by the calculation of energy mentioned in the current study. It warrants further evaluating the performance that our proposed IMO algorithm combines with Rosetta and i-TASSER for protein folding prediction in the future.

There are some limitations in the current study. The longest length of test sequence is 64 amino acids and it has 6^64^ possible combinations in 2D triangular lattice model with six neighbors, showing superior to other test algorithms [9, 21–23]. However, the performance of our proposed IMO algorithm is only based on 28 test data sets. It warrants further evaluating for more data sets and longer length of test protein sequences. It is noted that our proposed IMO method is based on the relative energy. For precise comparison, the absolute free energy for protein folding structure warrants further investigation in the future.

## Conclusions

This study uses an ab initio technique (hydrophobic polar model) to predict protein structures. This is one of the most commonly applied methods for protein structure prediction. We propose and develop here a combination of the IMO with a greedy algorithm for protein folding predictions assuming a hydrophobic polar model. Experimental results show that our proposed IMOG method can reliably seek and find the best solution among short sequences, and also effectively obtain satisfying results with longer sequences. Taken together, these results demonstrate that the hybrid algorithm, combining the IMO algorithm with a greedy algorithm provides a useful tool for protein folding predictions.
